# *Triplophysa anshuiensis*, a new species of blind loach from the Xijiang River, China (Teleostei, Nemacheilidae)

**DOI:** 10.3897/zookeys.744.21742

**Published:** 2018-03-20

**Authors:** Tie-Jun Wu, Mu-Lan Wei, Jia-Hu Lan, Li-Na Du

**Affiliations:** 1 Guangxi Institute of Fisheries, Nanning 530021, China; 2 Du’an Fishery Technique Popularization Station, Du’an 530700, China; 3 Kunming Institute of Zoology, Chinese Academy of Sciences, Kunming 650223, China

**Keywords:** cave fish, Guangxi, new species, *Triplophysa*

## Abstract

A new cave-dwelling fish, *Triplophysa anshuiensis*, is described here based on specimens collected from a karst cave in Guangxi Zhuang Autonomous Region, China, interconnected with the Hongshui River system, a tributary of the Xijiang River in the Pearl River (Zhu Jiang) Drainage. The species can be distinguished from its congeners by a combination of morphological characters. A key to the cave-dwelling species of *Triplophysa* in the Xijiang River is provided.

## Introduction

*Triplophysa* is an ecologically important and taxonomically challenging genus, distributed in lakes, rivers, and streams of the Qinghai-Tibet Plateau and adjacent region. The genus is diagnosed by a marked sexual dimorphism. In males the dorsal surface of the outer pectoral-fin rays are thickened, broadened, and covered by breeding tubercles; breeding tubercles are also present on the sides of the head, extending from the eye almost to the insertion of the maxillary barbels. Even though *Barbatula* species share the same sexual dimorphism, *Triplophysa* can be distinguished by close together nostrils in contrast to widely separated nostrils *in Barbatula* (Bănărescu and Nalbant 1968, Zhu 1989, Ren et al. 2012).

The distribution area of the genus extends westward to the Aral Sea Basin and interior drainages of Baluchistan and north-westward to western Mongolia and Republic of Tuva in Russia (Zhu 1989). In China, in addition to the Qinghai-Tibetan Plateau and Inner Mongolia, *Triplophysa* also occurs in Beijing, Shanxi, Sichuan, Yunnan, Chongqing, Hunan, Hubei, Guizhou, and Guangxi (Chen et al. 2009). The Guangxi Zhuang Autonomous Region lies in southern China where karst caves and subterranean streams are a dominant geological feature. The diversity of species is very high in many isolated rivers, especially in caves. So far, 27 cave-dwelling species in the genus *Triplophysa* have been described in China (Romero et al. 2009, Kottelat 2012, Lan et al. 2013, Yang et al. 2016, Li and Li 2017, Li and Lan 2017, Liu et al. 2017). According to Lan et al. (2013), these species can be placed into three groups according to their eye development, viz. normal eyes, reduced eyes, or no eyes. Of the 27 species, 23 are recorded from Xijiang River. Among them, ten species belong to the group with normal eyes, including *T. aluensis* Li & Zhu, 2000, *T. flavicorpus* Yang, Chen & Lan, 2004, *T. huapingensis* Zheng, Chen & Yang, 2012, *T. longipectoralis* Zheng, Du, Chen & Yang, 2009, *T. nandanensis* Lan, Yang & Chen, *T. nasobarbatula* Wang & Li, 2001, *T. tianxingensis* Yang, Li & Chen, 2016, *T. xiangshuingensis* Li, 2004, *T. yunnanensis* Yang, 1990, and *T. zhenfengensis* Wang & Li, 2001. Five species belong to the group with reduced eyes, namely *T. langpingensis* Yang, 2013, *T. luochengensis* Li, Lan, Chen & Du, 2017, *T. macrocephala* Yang, Wu & Yang, 2012, *T. tianeensis* Chen, Cui & Yang, 2004, and *T. tianlinensis* Li, Li & Lan, 2017. The group without eyes includes eight species, namely, *T. dongganensis* Yang, 2013, *T. fengshanensis* Lan, 2013, *T. gejiuensis* Chu & Chen, 1979, *T. huanjiangensis* Yang, Wu & Lan, 2011, *T. lihuensis* Wu, Yang & Lan, 2012, *T. longibarbata* Chen, Yang, Sket & Aljancic, 1998, *T. qiubeiensis* Li & Yang, 2008, and *T. shilinensis* Chen & Yang, 1992 (Zheng et al. 2012, Lan et al. 2013, Zhang and Zhao 2016, Li and Li 2017, Li and Lan 2017). In May 2012, two specimens of nemacheiline loach were collected from a karst cave in Anshui Village, Lingyun County, Guangxi Zhuang Autonomous Region, China. These specimens represent a new species of *Triplophysa*, which is described herein.

## Materials and methods

Specimens were preserved in 8 % formalin and are maintained at the Kunming Natural History Museum of Zoology, Kunming Institute of Zoology (KIZ), Chinese Academy of Sciences. Counts and measurements follow Kottelat (1990), except for the median caudal-fin length, which is the length of the shortest branched caudal-fin ray. Measurements were made point-to-point with digital calipers recorded to 0.1 mm. Abbreviations: *P_L_*-*A*, distance between pelvic-fin origin and anal-fin origin; *P_T_*-*P_L_*, distance between pectoral-fin origin and pelvic-fin origin.

Data on *T. huanjiangensis*, *T. fengshanensis*, and *T. dongganensis* are cited from Lan et al. (2013). Data on *T. xiangshuingensis* and *T. zhenfengensis* are from Li (2004) and Wang and Li (2001), respectively. Other comparative species were measured at KIZ, Chinese Academy of Sciences.

## Results

### Triplophysa anshuiensis sp. n.

Taxon classificationAnimaliaCypriniformesNemacheilidae

http://zoobank.org/4E1C8A91-8F51-46F2-B66D-99B9F7C98EAB

**Figure 1.**
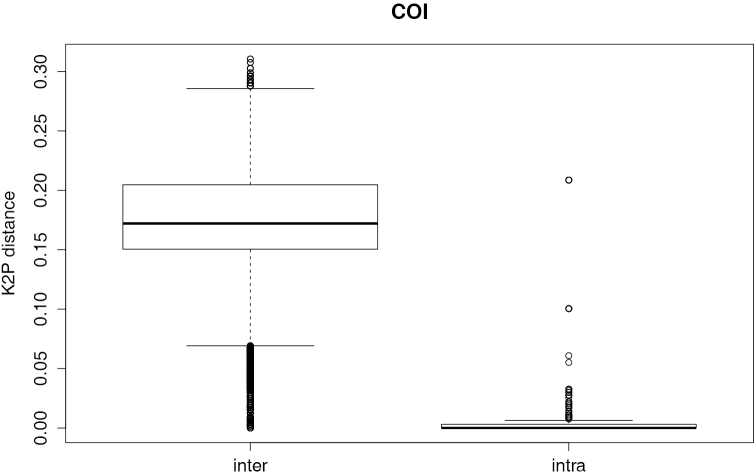
Lateral and ventral views of *Triplophysa anshuiensis* sp. n., holotype KIZ 2012005747, 65.2 mm SL. Scale = 1 cm.

**Figure 2.**
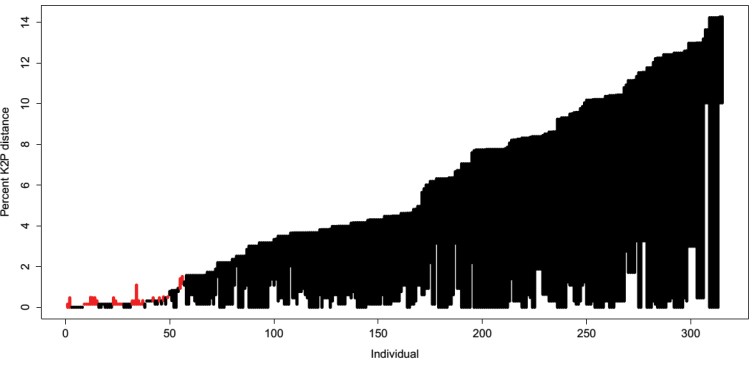
Type locality of *Triplophysa anshuiensis* sp. n., a cave in Anshui Village, Guangxi, China.

**Figure 3.**
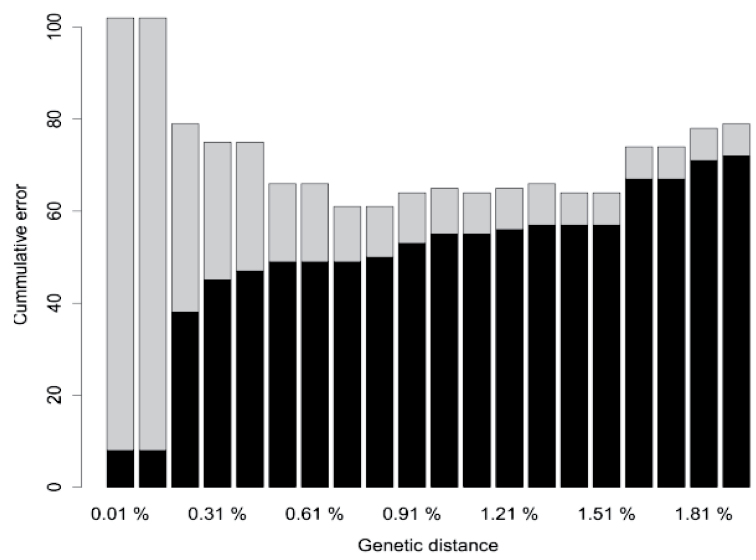
Posterior chamber of air bladder of *Triplophysa anshuiensis* sp. n.

Figures 1 , 2 , 3 ; Table 1

#### Type specimens.

***Holotype***. Kunming Natural History Museum of Zoology, Kunming Institute of Zoology, Chinese Academy of Sciences, Kunming, KIZ2012005747, 65.2 mm standard length, Anshui Village, Lingyun County, Guangxi Zhuang Autonomous Region, China; 24.3632N, 106.7412E, Altitude 719 m; collected by J. H. Lan, 12 May 2012. ***Paratypes.*** Kunming Natural History Museum of Zoology, KIZ 2012005746, 68.5 mm SL; collected with holotype.

#### Diagnosis.

*Triplophysa anshuiensis* can be distinguished from all species of *Triplophysa* by the following combination of characters: eyes absent, gill rakers absent in outer row and eight gill rakers in inner row on first gill arch, 14 branched caudal-fin rays, body scaleless, tips of depressed pectoral fins not reaching pelvic-fin origin, 12–13 preoperculo-mandibular pores, lateral line complete, posterior chamber of air bladder developed.

#### Description.

Morphometric data of type specimens of *Triplophysa anshuiensis* are given in Table 1. D, 4/7–8; A, 2/6; P, 1/10; V, 1/6, C, 14; 8 inner gill rakers in inner row on first gill arch (n=1). Cephalic lateral-line canals with 2+2 supra-temporal, 8 supraorbital, 4+8–9 infraorbital, and 12–13 preoperculo-mandibular pores. Lateral line complete, with 52–54 pores.

**Table 1.** Morphometric data of type specimens of *Triplophysa anshuiensis* sp. n.

Measurements	Paratype	Holotype
	2012005746	201005747
Standard length	68.5	65.2
Lateral head length	14.5	13.8
**Percent of SL**		
Body depth	15.7	12.2
Lateral head length	21.1	21.1
Predorsal length	52.6	47.5
Prepelvic length	55.1	52.0
Preanal length	79.2	72.5
Preanus length	71.4	66.9
Caudal-peduncle length	15.1	14.6
Caudal-peduncle depth	9.0	9.8
**Percent of HL**		
Maximum head depth	50.7	47.7
Maximum head width	53.1	52.0
Pectoral fin length/Pt-Pl	66.4	59.8
Pelvic fin length/Pl-A	77.2	63.6
CPD/CPL	59.2	67.3

*Body* elongated, slightly compressed anteriorly, more strongly compressed posteriorly. Deepest point of body in front of dorsal-fin origin, body depth 12–16% of SL. Head compressed, maximum width greater than depth. Anterior and posterior nostrils adjacent, anterior nostril in short tube with elongated barbel-like tip, tip of nostril barbel reaching posterior margin of posterior nostrils. Eyes absent. Mouth inferior, mouth corner situated below anterior nostril. Lips thick with shallow furrows; lower lip with a “V” type median notch. Upper and lower jaw arched, processus dentiformis on upper jaw absent and no corresponding notch on lower jaw. Three pairs of barbels; inner rostral barbel 50–54 % of lateral head length; outer rostral barbel 20–26 % of lateral head length; maxillary barbel 36–39 % of lateral head length. Body scaleless. Intestine straight. Posterior chamber of air bladder developed, reaching dorsal-fin origin (Fig. 3).

*Dorsal fin* distally truncate, origin anterior to pelvic-fin insertion, situated slightly anterior to midpoint between tip of snout and caudal-fin base; first branched ray longest, reaching anus when adpressed vertically. Anal fin distally truncate. Pectoral fins moderately developed, 60–67 % of distance between pectoral and pelvic fins. Tip of depressed pelvic fin reaching anus. Anus short distance from anal-fin origin. Caudal fin forked, tips pointed.

*Coloration.* Fixation in 8 % formalin, body yellowish. Black pigments irregularly present on dorsum of body.

*Sexual dimorphism.* No sexual dimorphism was observed in the two specimens.

#### Distribution.

A karst cave in Anshui Village, Lingyun County, Guangxi Zhuang Autonomous Region, China, whichis interconnected with the Hongshui River system, a tributary of the Xijiang River in the Pearl River (Zhu Jiang) Drainage.

#### Habitat and ecology.

An underground stream was found about 40 m from and 10 m below the entrance to a cave. *Triplophysa anshuiensis* inhabits pools in which the substratum is mud and cobblestones (Fig. 2). Pools are interconnected by underwater channels. No other species were recorded in this cave.

#### Etymology.

The specific name, *anshuiensis*, is derived from the village Anshui, the type locality of the species.

## Discussion

Thirteen *Triplophysa* species have been recorded in karst caves and subterranean streams in the Guangxi Zhuang Autonomous Region (Yang et al. 2004, Lan et al. 2013): *T. dongganensis*, *T. fengshanensis*, *T. flavicorpus*, *T. huanjiangensis*, *T. huapingensis*, *T. langpingensis*, *T. lihuensis*, *T. longipectoralis*, *T. luochengensis*, *T. macrocephala*, *T. nandanensis*, *T. tianeensis*, and *T. tianlinensis*. In morphology, the new species can be distinguished from *T. huapingensis*, *T. longipectoralis*, *T. nandanensis*, and *T. flavicorpus* by the eyes absent (vs. normally developed). Furthermore, *T. anshuiensis* can be easily distinguished from *T. flavicorpus* by the gill rakers absent in outer row on first gill arch (vs. 5–6). The new species can be distinguished from *T. nandanensis* by the posterior chamber of the air bladder developed (vs. reduced). *Triplophysa anshuiensis* differs from *T. huapingensis* and *T. longipectoralis* by 8 gill rakers in inner row on first gill arch (vs. 13), 14 branched caudal-fin rays (vs. 16 in *T. huapingensis*), the tip of the pectoral fin when depressed not reaching pelvic-fin origin (vs. exceeding the pelvic-fin origin in *T. longipectoralis*). Among the Guangxi species with the eyes degenerated or absent, the new species can be distinguished from *T. langpingensis*, *T. luochengensis*, *T. macrocephala*, *T. tianeensis*, and *T. tianlinensis* by the eyes absent (vs. degenerated, with black pigment). From *T. luochengensis*, it can be further distinguished by 14 branched caudal-fin rays (vs. 15–17) and a scaleless body (vs. scaled), from *T. tianeensis* by 14 branched caudal-fin rays (vs. 15–17), 8 gill rakers in inner row on first gill arch (vs. 10–11), and posterior chamber of air bladder developed (vs. reduced), from *T. macrocephala* and *T. tianlinensis* by 14 branched caudal-fin rays (vs. 15–17), and from *T. langpingensis* by a complete lateral line (vs. incomplete).

In the group of Guangxi species with completely reduced eyes, *T. anshuiensis* is similar to *T. lihuensis*, *T. huanjiangensis*, *T. fengshanensis*, and *T. dongganensis* in having no scales on the whole body. The new species can be distinguished from *T. lihuensis* and *T. huanjiangensis* by 8 gill rakers in inner row on first gill arch (vs. 10–13 in *T. lihuensis* and *T. huanjiangensis*, whereas unknown in *T. dongganensis* and *T. fengshanensis*), from *T. fengshanensis* by 14 branched caudal-fin rays (vs. 16), and caudal peduncle depth 6.6–6.9 times in standard length (vs. 5.0–5.3), and from *T. dongganensis* by caudal peduncle depth 10.2–11.2 times in standard length (vs. 11.4–16.9), and caudal peduncle depth 1.5–1.7 times in its length (vs. 1.8–2.9). In addition to the species of *Triplophysa* from Guangxi, there are ten more troglobitic *Triplophysa* species recorded in the Xijiang River, including *T. aluensis*, *T. gejiuensis*, *T. longibarbata*, *T. nasobarbatula*, *T. qiubeiensis*, *T. shilinensis*, *T. xiangshuingensis*, *T. yunnanensis*, and *T. zhenfengensis* (Chen 1992, Chu and Chen 1979, Wang and Li 2001, Chen and Yang 2005, Yang et al. 2016, Liu et al. 2017). *Triplophysa anshuiensis* can be easily distinguished from *T. nasobarbatula*, *T. xiangshuingensis*, *T. yunnanensis*, and *T. zhenfengensis* by eyes absent (vs. normal), body colorless (vs. body with color pattern), scaleless (vs. scaled in *T. nasobarbatula*, *T. xiangshuingensis*, *T. yunnanensis*, and *T. zhenfengensis*), and 14 branched caudal-fin rays. *Triplophysa anshuiensis* can be distinguished from other species by the following characters: eyes absent (vs. degenerated, with black pigment in *T. aluensis*), 8 supraorbital pores (vs. 5 in *T. gejiuensis* and absent in *T. shilinensis*), 12–13 preoperculo-mandibular pores (vs. 7 in *T. gejiuensis* and absent in *T. shilinensis*), and caudal peduncle length 1.5–1.7 times in its length (vs. more than two times in *T. qiubeiensis* and *T. longibarbata*).

### Key to cave-dwelling species of *Triplophysa* in Xijiang Drainage

**Table:** 

1	Eyes normal	**2**
–	Eyes reduced or absent	**10**
2	Body scaleless	**3**
–	Body covered by scales	**4**
3	Fourteen branched caudal-fin rays	***T. xiangshuingensis***
–	Sixteen branched caudal-fin rays	***T. aluensis***
4	Processus dentiformis present in upper jaw	***T. zhenfengensis***
–	Processus dentiformis absent in upper jaw	**5**
5	Tip of pectoral fin exceeding pelvic fin origin	***T. longipectoralis***
–	Tip of pectoral fin not reaching pelvic fin origin	**6**
6	Tip of depressed pelvic fin exceeding anus	***T. flavicorpus***
–	Tip of depressed pelvic fin not reaching anus	**7**
7	Eye diameter / lateral head length < 10 %	***T. yunnanensis***
–	Eye diameter / lateral head length > 10 %	**8**
8	Caudal peduncle length/depth 1.8–2.1	***T. nasobarbatula***
–	Caudal peduncle length/depth 1.2–1.7	**9**
9	Pectoral fin length / *P_T_*-*P_L_* 58–69 %	***T. huapingensis***
–	Pectoral fin length / *P_T_*-*P_L_* 78–82 %	***T. nandanensis***
10	Eyes reduced	**11**
–	Eyes absent	**15**
11	Lateral line incomplete	***T. langpingensis***
–	Lateral line complete	**12**
12	Body covered by scales	***T. luochengensis***
–	Body scaleless	**13**
13	Nine gill rakers in inner row on the first gill arch…	***T. macrocephala***
–	Ten–11 gill rakers in inner row on the first gill arch	**14**
14	Eight–9 branched pectoral-fin rays	***T. tianeensis***
–	Ten branched pectoral-fin rays	***T. tianlinensis***
15	Lateral-line absent	**16**
–	Lateral-line complete	**17**
16	Posterior chamber of air bladder developed	***T. huanjiangensis***
–	Posterior chamber of air bladder reduced	***T. lihuensis***
17	Six branched dorsal-fin rays	***T. shilinensis***
–	Seven branched dorsal-fin rays	**18**
18	Five branched pelvic-fin rays	***T. qiubeiensis***
–	Seven branched pelvic-fin rays	**19**
19	Sixteen branched caudal-fin rays	***T. fengshanensis***
–	Thirteen–15 branched caudal-fin rays	**20**
20	Caudal peduncle length/depth 3–3.1	***T. longibarbata***
–	Caudal peduncle length/depth less than 3	**21**
21	Standard length/caudal peduncle depth more than 14	***T. dongganensis***
–	Standard length/ caudal peduncle depth less than 12	**22**
22	Cephalic lateral-line canals with 8 supraorbital and 12–13 preoperculo-mandibular pores	***T. anshuiensis* sp. n.**
–	Cephalic lateral-line canals with 5 supraorbital and 7 preoperculo-mandibular pores	***T. gejiuensis***

### Comparative material

All specimens from Pearl River.

*Triplophysa gejiuensis*: KIZ 7803001, holotype, 44.0 mm SL, 7803002–005, paratypes, 4 ex., 42.7–46.3 mm SL, Gejiu County, Yunnan Province.

*T. huapingensis*: KIZ 2008007607, holotype, 62.3 mm SL; KIZ 2008007606, 2008007608–610, paratypes, 4 ex, 44.5–59.3 mm SL, Huaping Town, Leye County, Guangxi Zhuang Autonomous Region.

*T. macrocephala*: KIZ 04100631, holotype, 55.7 mm SL; KIZ 04100618–619, paratypes, 3 ex, 49.3–53.8 mm SL, Lihu County, Nadan City, Guangxi Zhuang Autonomous Region.

*T. shilinensis*: KIZ 2004013853–854, 2 ex, 41.5–46.7 mm SL, Shilin County, Yunnan Province.

*T. tianeensis*: KIZ 200301003, holotype, 57.9 mm SL; KIZ 200301001–002, KIZ 200301004–006, paratypes, 5 ex, 35.5–59.1 mm SL, Tian’e County, Guangxi Zhuang Autonomous Region.

*T. nandanensis*: KIZ 911911, holotype, 58.2 mm SL, KIZ 911008–009, 9110012–017, paratypes, 9 ex, 36.9–81.3 mm SL, Liuzai County, Nandan City, Guangxi Zhuang Autonomous Region.

*T. longipectoralis*: KIZ 01050218, holotype, 50.7 mm SL; KIZ 01050219–221, 01050223–224, paratypes, 5 ex, 36.9–52.0 mm SL, Huanjiang County, Hechi City, Guangxi Zhuang Autonomous Region.

*T. lihuensis*: KIZ 2010003082, holotype, 59.3 mm SL; KIZ 2010003083–084, paratypes, 2 ex, 55.6–58.3 mm SL, Renguang Village, Lihu Town, Nandan County, Guangxi Zhuang Autonomous Region.

*T. langpingensis*: uncat. 3 ex, 44.7–70.9 mm SL, Langping County, Guangxi Zhuang Autonomous Region.

*T. aluensis*: KIZ 20006005–007, 3 ex, 43.2–82.6 mm SL, A’lu County, Yunnan.

*T. yunnanensis*: KIZ 874200, holotype, 59.8 mm SL, KIZ 874197, KIZ 874199, paratypes, 2 ex, 48.1–62.2 mm SL, Jiuxiang Town, Yiliang County, Yunnan Province.

*T. nasobarbatula*: KIZ 2005001276, KIZ 2005001325, 2 ex, 34.2–40.5 mm SL, Libo County, Guizhou Province.

## Supplementary Material

XML Treatment for Triplophysa anshuiensis
